# The Role of Natural Enemy Foraging Guilds in Controlling Cereal Aphids in Michigan Wheat

**DOI:** 10.1371/journal.pone.0114230

**Published:** 2014-12-04

**Authors:** Shahlo Safarzoda, Christine A. Bahlai, Aaron F. Fox, Douglas A. Landis

**Affiliations:** Department of Entomology, Michigan State University, East Lansing, Michigan, United States of America; Natural Resources Canada, Canada

## Abstract

Insect natural enemies (predators and parasitoids) provide important ecosystem services by suppressing populations of insect pests in many agricultural crops. However, the role of natural enemies against cereal aphids in Michigan winter wheat (*Triticum aestivum* L.) is largely unknown. The objectives of this research were to characterize the natural enemy community in wheat fields and evaluate the role of different natural enemy foraging guilds (foliar-foraging versus ground-dwelling predators) in regulating cereal aphid population growth. We investigated these objectives during the spring and summer of 2012 and 2013 in four winter wheat fields on the Michigan State University campus farm in East Lansing, Michigan. We monitored and measured the impact of natural enemies by experimentally excluding or allowing their access to wheat plants infested with *Rhopalosiphum padi* (L.) and *Sitobion avenae* (F.) (Hemiptera: Aphidae). Our results indicate that the natural enemy community in the wheat fields consisted mostly of foliar-foraging and ground-dwelling predators with relatively few parasitoids. In combination, these natural enemy groups were very effective at reducing cereal aphid populations. We also investigated the role of each natural enemy foraging guild (foliar-foraging versus ground-dwelling predators) independently. Overall, our results suggest that, in combination, natural enemies can almost completely halt early-season aphid population increase. Independently, ground-dwelling predators were more effective at suppressing cereal aphid populations than foliar-foraging predators under the conditions we studied. Our results differ from studies in Europe and the US Great Plains where foliar foraging predators and parasitoids are frequently more important cereal aphid natural enemies.

## Introduction

Cereal aphids (Hemiptera: Aphididae) are serious pests of grain crops worldwide [1]–[3], causing economic damage directly by feeding on the plants and indirectly by transmitting cereal and barley yellow dwarf viruses [4]–[8]. Both chemical and biological methods have been used to manage aphids and reduce the spread of viruses in cereals. While effective in aphid control, intensive use of insecticides can lead to increased production costs [9], development of insecticide resistance, increased aphid movement from plant to plant increasing virus spread [10]–[12] and negative effects on human health and the environment [13], [14]. Insecticides can also reduce the abundance and diversity of predatory insects that regulate aphid populations [15]–[18]. Fostering natural enemies in an early-season crop like winter wheat, *Triticum aestivum* (L.) may have important implications for pest suppression in later season crops like corn and soybean, as mobile natural enemies may move to adjacent crops after early-season crops are harvested [19]. In spite of this, prophylactic use of insecticides on wheat is common [20].

Pest suppression by natural enemy communities is an important ecosystem service [21]–[23]. Naturally occurring enemies that prey on aphids can prevent populations from multiplying beyond economic thresholds and prevent yield loss [24]–[26] thereby reducing the need for insecticide use. Reducing chemical inputs can in turn increase populations of beneficial insects. For example, a study of organic and conventional wheat fields found the abundance of natural enemies and relative aphid control was higher in fields with no pesticide treatments [27]. Östman et al. [21] showed that *Rhopalosiphum padi* (L.) establishment was rarer in organic compared to conventional fields which could be a result of higher numbers of natural enemies in the organic fields. In a European study, cereal aphid populations were 172% higher on wheat plants when all natural enemies were excluded [25].

Cereal aphid predators and parasitoids can be coarsely grouped into two foraging guilds: foliar foraging and ground-dwelling natural enemies [28]. Foliar-foraging natural enemies include Coccinellidae (Coleoptera), Chrysopidae (Neuroptera), Syrphidae (Diptera), and parasitoid wasps (Hymenoptera). These taxa typically forage in the upper vegetation of cereal plants, often fly between plants while foraging, and predominantly feed on aphids [29]. Ground-dwelling generalist predators include Carabidae (Coleoptera), Staphylinidae (Coleoptera), and Araneae (spiders). These enemies live and forage near the ground and may include diverse prey in their diets, including aphids [30]. Ground-dwelling predators primarily prey on aphids occurring on the lower portion of the plant, or that have fallen from the plant due to disturbance. Both groups of natural enemies, acting independently or together, can reduce aphid population density [26], [30], in turn reducing plant damage and increasing yield [22].

Foliar-foraging and ground-dwelling predators can interact, suppressing aphid populations to greater extent than when they act independently [25], [31]. For example, Losey and Denno [32] found that in alfalfa in the absence of foliar-foraging predators, ground-dwelling predators had a very small effect on aphid populations. However, when foliar-foraging Coccinellidae predators were added to the system, the effect of both predator groups on aphid suppression was higher than the sum of each community. The reason for this synergetic effect was that Coccinellidae foraging caused aphids to drop from the vegetation onto the ground, where they were consumed by Carabidae [32].

While the complex of aphid natural enemies in cereal crops has been studied in parts of North America [33] e.g. South Dakota [7], eastern Washington [34], [35], and Colorado [2], [36], to our knowledge, the community structure or impacts of cereal aphid enemies has not been described in Michigan or the Midwest United States. To investigate the role of natural enemies in controlling cereal aphid populations in this region we conducted exclusion cage field studies in 2012 and 2013 in East Lansing, Michigan. The overall goals of these studies were to characterize the natural enemy community in Michigan winter wheat fields, determine their community-wide effect on aphid population growth, and determine the relative impact of foliar-foraging versus ground-dwelling predators on suppression of cereal aphid populations.

## Methods

### Study sites

Two independent studies were conducted on the Michigan State University campus, East Lansing, Michigan in 2012–13 using natural enemy exclusion cages to explore the impact of natural enemy communities on cereal aphid population growth. The first study was conducted in four different winter wheat fields in 2012 (2 fields) and 2013 (2 fields). The second study was conducted only in 2013 using the same sites (2 fields) as the first study. For both studies, the wheat was sown in October of the prior year and utilized for experimentation in May of the following year. Each field received typical commercial applications of herbicides, fungicides and fertilizers at rates determined by the university farm manager (**Table 1**). No insecticides were applied in the fields in either year. In each field, a 30×20 m area was delineated at least 35 m from the field edge. Individual plots within this area were established 5 m equidistant from each other in a completely randomized design.

**Table 1:** Agronomic records at study sites.

Study site	Year	Area (ha)	Cultivar	Seeding	Fungicide(mg/ha)**	Fertilizer46-0-0 (kg/ha)
Field 1	2012	8.7	Ruby Red	10/5/2011	94.6	73.9
Field 2	2012	18.5	Ruby Red	10/18/2011	94.6	181.0
Field 3	2013	12.1	Red Devil	10/4/2012	0	41.3
Field 4	2013	9.5	Ruby Red	10/17/2012	0	83.5

*DuPont Thifensulfuron-methyl–25%, Tribenuron- methyl- 25%, other ingredients–50%.

**Stratego YLD -BAYER Prothioconazole–10.8%, Trifloxystrobin, –32.3%, other ingredients–56.9%.

Michigan State University campus farm fields used for cereal aphid and natural enemy studies, East Lansing, Michigan, 2012–2013. All plots also received a single application of Affinity Broadspec* at 28.0 g/ha.

### Study 1: Community-wide Natural Enemy Impacts

**Figure 1:**
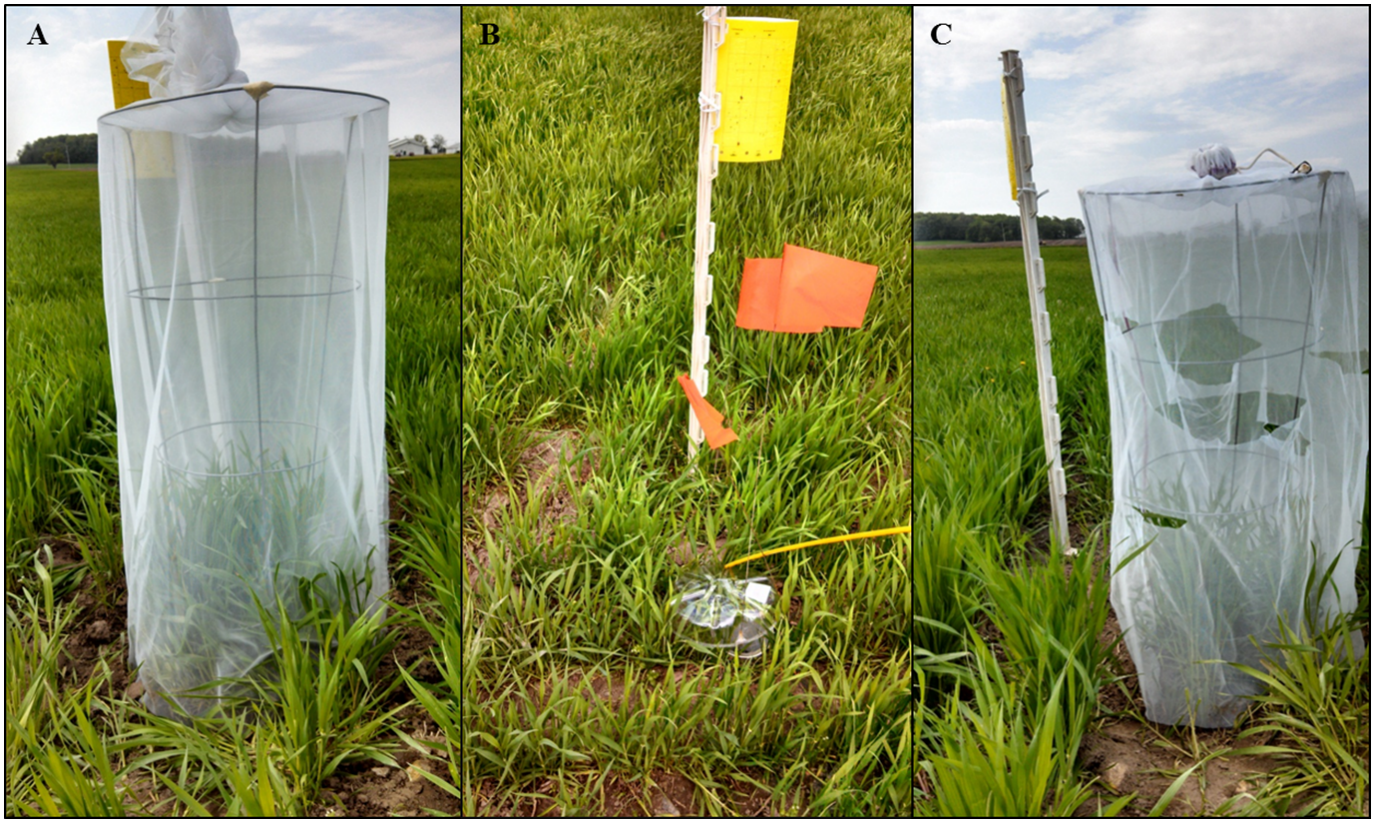
Photo illustrating natural enemy community exclusion treatments used in Study 1. A) closed plot caged to exclude all natural enemies, B) open plot allowing access to all natural enemies and C) sham plots, which are caged but, with holes on the ground and canopy levels to allow access by natural enemies.

The first study contrasted cereal aphid population growth using; open plots, which allowed unrestricted access of the ambient natural enemy community, closed plots which attempted to exclude all natural enemies, and sham cages which allowed natural enemy access but controlled for cage effects. Open plots consisted of circular area with a diameter of 0.36 m, without any barrier to natural enemies. Closed plots consisted of 1 m tall, 0.36 m diameter wire tomato support cages (**Figure 1**), covered with sewn sleeves of no-see-um mesh (approx. 625 holes per 6.45 cm^2^, Skeeta Inc., Bradenton, FL). The bottoms of the cages were buried 15 cm into the ground to prevent access by ground-dwelling predators. The top of the sleeves were tied with nylon cord to prevent flying predators from entering. The sham cages were identical to closed plots with the exception that the sleeves had multiple 10 cm slits on each side and at the bottom of the mesh allowing entry of predators and parasitoids, including foliar-foraging and ground-dwelling natural enemies. Cage treatments were initiated on May 18, 2012 and May 14, 2013, and were replicated five times per field in a completely randomized design.

### Study 2: Guild-specific Natural Enemy Impacts

**Figure 2:**
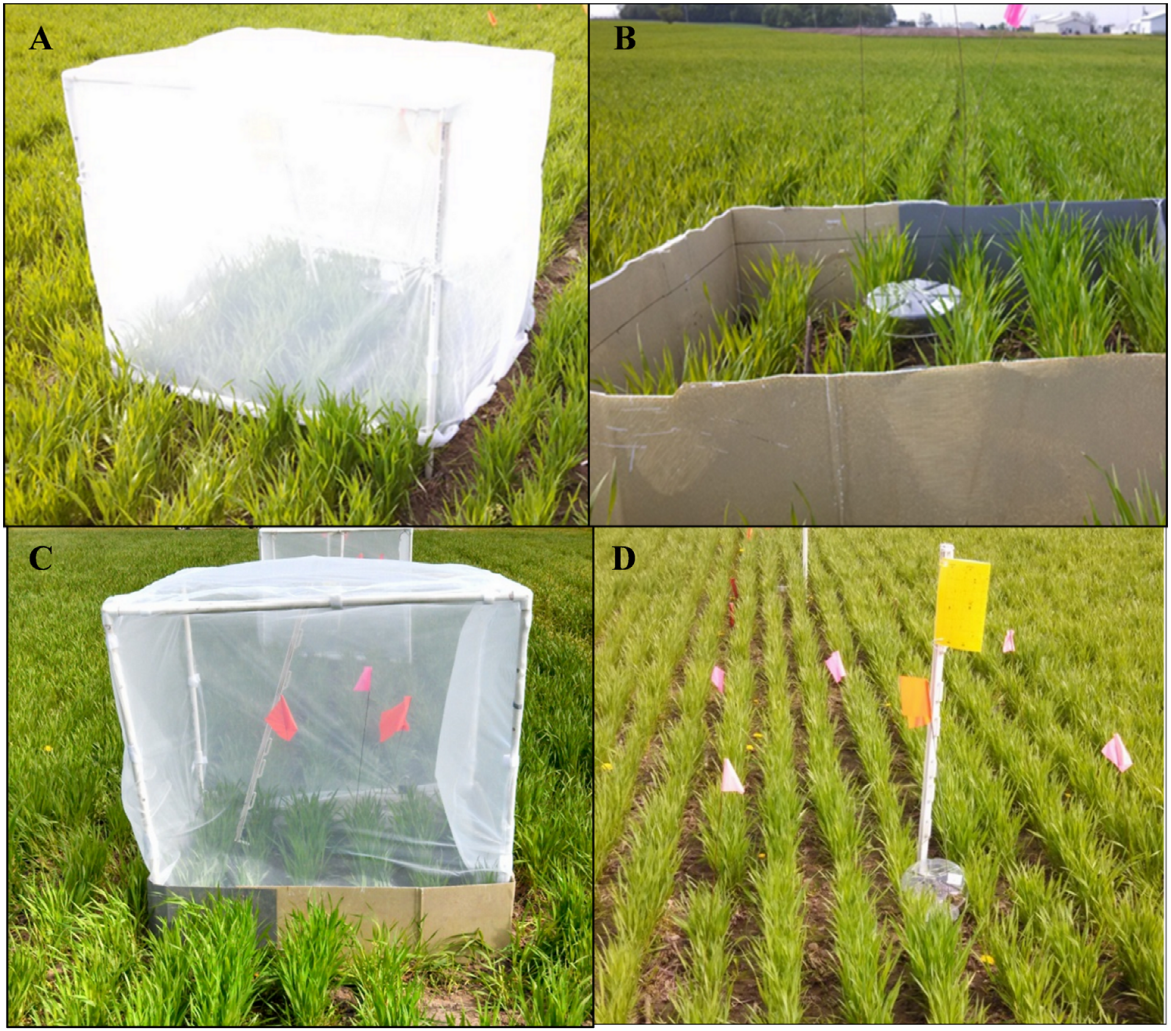
Photo illustrating the natural enemy guild exclusion treatments used in Study 2. Each plot was assigned a cage treatment to exclude different groups of natural enemies from aphid populations. Treatments included exclusion of **A**) foliar-foraging predators and parasitoids (-F), **B**) ground-dwelling predators (-G), **C**) and all natural enemies (-F-G), and **D**) open plot (O), which allowed access to all natural enemies and served as a control.

The second study contrasted cereal aphid population growth in the presence of selected natural enemy guilds in 1×1 m plots. Treatments included: exclusion of foliar-foraging predators and parasitoids (hereafter called -F), exclusion of ground-dwelling predators (-G), exclusion of both foliar and ground-dwelling natural enemies (-F-G), and fully open plots (O), which were exposed to all natural enemies. Each of the four treatments consisted of a 1 m^3^ PVC frame cage, erected around the plots, with the legs buried in the soil. In the plots excluding ground-dwelling predators (-G), and in the plots excluding all natural enemies (-F-G), a 30 cm tall corrugated plastic barrier was erected around the PVC frame. The bottom 10 cm of this barrier was buried in the soil so that 20 cm was left above ground to restrict access by ground-dwelling predators (**Figure 2A, B**). To exclude all natural enemies (-F-G) and foliar-foraging predators and parasitoids (-F), the top and all sides of the PVC frame were additionally covered with no-see-um mesh, to prevent flying predators from entering the cage. To exclude all natural enemies (-F-G), the bottoms of the mesh were buried 5 cm into the ground while in -F plots, the bottoms of the mesh were raised 2 cm above the ground to allow access by ground-dwelling predators (**Figure 2C**). Finally, open plots (O) consisted of 1×1 m area demarcated with flags, without any barrier to natural enemies (**Figure 2D**). All treatments were initiated on May 14, 2013 and were replicated five times in each field.

### Natural enemy sampling

To characterize the overall natural enemy community in the wheat fields, we used three sampling methods in both studies. Natural enemies were sampled weekly (Study 1 from May 29 to June 19 of 2012 and May 21 until June 5 2013, and Study 2 from May 21 until June 5 of 2013). To sample the ground-dwelling predator community, pitfall traps (plastic Solo cups, 11 cm in diameter and 14 cm in depth) were placed at each study site. Four pitfall traps per site were used for Study 1 and twenty per site for Study 2. Pitfall traps were filled 1/3 of the way with 40% propylene glycol solution. The pitfall traps for Study 1 were established next to the plots and 4 m apart from one another, and for Study 2 were established inside the cages 5 m apart from one another. To sample foliar foraging predators, 23×28 cm yellow sticky card traps (PHEROCON AM, Great Lakes IPM, Vestaburg, Michigan) were placed next to the pitfall traps (Study 1: n = 4; Study 2: n = 20). The yellow sticky cards were hung on a plastic step-in fence posts (Zareba Systems, Lititz, PA), and positioned just above the plant canopy. Finally, on each sample date, natural enemies were counted by visual observation for a fixed time (five minutes) in each plot. Any natural enemies found in closed plots were manually removed during the sampling. All the predators were identified in the field or returned to the laboratory for identification. Araneae and Opiliones were identified to order while most other organisms were identified to family. Due to their potential importance in aphid control, Coccinellidae were identified to species. Coccinellidae that were difficult to identify in the fields or were missing identifying features on sticky cards were categorized as “other Coccinellidae.” The overall mean abundance (± SEM) of each natural enemy taxa was calculated by field and by year.

### Aphid Infestation and Population Growth

On May 18, 2012 and May 14, 2013 all plots were infested with 50 (Study 1) and 100 (Study 2) laboratory reared *R. padi* of mixed adult and nymphal stages, when wheat plants in the field were at the six-seven Feekes growth stage [37], [38]. In 2012, the initial establishment of *R. padi* in the plots failed so all plots were reinfested with 50 additional aphids on May 23. Prior to aphid infestation, natural enemies were removed from each plot by hand and vacuuming the plot area using a modified leaf blower [39]. The aphids were transferred to the field treatment plants by cutting leaves infested with aphids from the cultured greenhouse plants and placing them between the leaves of the middle wheat plant of the plot. Aphid abundance was assessed once per week after infestation, by counting all aphids on all plants within the plots and recording number of wheat tillers present in the plots. Both alate and apterous aphids were recorded. On the first sampling date, naturally occurring *S. avenae* were also observed, so from that date on, counts of *S. avenae* were also recorded. To control for sampling effects, plants were manipulated to the same degree in all plots to ensure that all plants, aphids, and natural enemies received the same amount of disturbance.

### Statistical analyses

For both studies, ANOVA procedures (R version 3.0.2) [40] were used to analyze within year populations of each species of per tiller of wheat by cage treatment, site, and sample day. A negative binomial error structure was used in all ANOVA analyses, and Rao’s efficient score test was used in lieu of the F statistic because it is appropriate for models with non-normal error structure [41]. If statistically significant treatment differences were observed, means were compared by pairwise t-tests that had been Holm-adjusted for multiple comparisons to compare treatments. The code used for analysis, in the form of an R script, as well as all data used in analysis are available in a GitHub repository at https://github.com/cbahlai/Safarzoda_2014.

## Results

### Study 1: Community-wide Natural Enemy Impacts

Using all three sampling methods, we collected a total of 4,065 natural enemies representing 13 taxa (**Table 2**). Overall, seven taxa of ground-dwelling natural enemies were captured in pitfall traps over both years. In 2012, the most common family in Field 1 were Formicidae followed by Araneae, Carabidae and Opiliones. In contrast, in Field 2 the most common natural enemies were Araneae followed by Carabidae. In 2013, in Field 3 and Field 4 Carabidae were the most common taxa followed by Araneae and Staphylinidae.

**Table 2:** Natural enemy captures Study 1.

Collection method	2012						2013					
Pitfall trap	Field 1			Field 2			Field 3			Field 4		
Coccinellidae	0.2	±	0.1	0.7	±	0.2	0.1	±	0.1	1.2	±	0.5
Carabidae	9.1	±	1.7	4.6	±	1.2	14.9	±	3.5	12.8	±	2.1
Formicidae	30.7	±	10.2	2.6	±	0.8	2.9	±	1.2	7.6	±	1.9
Araneae	12.8	±	1.7	17.5	±	1.8	10.8	±	2.0	7.5	±	1.5
Opiliones	7.1	±	1.4	1.4	±	0.3	0.1	±	0.1	0.3	±	0.2
Elateridae	0.4	±	0.2		0		0.1	±	0.1	0.2	±	0.1
Staphylinidae	NA			NA			7.6	±	2.4	4.9	±	1.3
**Yellow sticky card**												
*C. maculata*	0.2	±	0.1	0.4	±	0.1	0.4	±	0.2	1.2	±	0.3
*H. convergens*	0.6	±	0.2	0. 6	±	0.3		0			0	
*C. septempunctata*	0.3	±	0.1	0.3	±	0.2		0		0.1	±	0.1
*H. axyridis*	0.4	±	0.2	2.0	±	0.4		0			0	
Other Coccinellidae	0.2	±	0.1	0.4	±	0.2	0.2	±	0.1	0.2	±	0.2
Dolichopodidae	2.9	±	1.1	3.1	±	0.7		0			0	
Chrysopidae	2.4	±	0.8	5.1	±	1.0	0.8	±	0.3	0.3	±	0.2
Syrphidae	1.7	±	0.5	10.1	±	1.6	4.8	±	1.4	4.0	±	1.0
Nabidae		0			0		1.2	±	0.8	0.3	±	0.2
Cantharidae	0.1	±	0.0	0.3	±	0.2	0.8	±	0.3	1.4	±	0.6
**Visual observation**												
*C. maculata*		0			0		0.3	±	0.1	0.5	±	0.1
*C. septempunctata*	0.3	±	0.1	0.1	±	0.0	0.1	±	0.1	0.3	±	0.1
*C. septempunctata* larvae	0.2	±	0.1	0.1	±	0.0		0			0	
*H. axyridis*	0.2	±	0.1	0.1	±	0.0	0.2	±	0.1	0.2	±	0.1
*H. axyridis* larvae	0.1	±	0.0		0		0.03	±	0.03	0.1	±	0.0
Other Coccinellidae larvae	0.2	±	0.1	0.3	±	0.1		0			0	
Chrysopidae larvae	0.1	±	0.0	0.2	±	0.1	0.1	±	0.1		0	
Syrphidae	0.3	±	0.1	0.3	±	0.1	0.2	±	0.1	0.3	±	0.1
Carabidae		0		0.1	±	0.0	0.2	±	0.1		0	
Araneae	0.1	±	0.1	0.6	±	0.2	0.1	±	0.0		0	
Anthocoridae	0.1	±	0.0	0.2	±	0.1	0.1	±	0.1	0.1	±	0.0

Mean numbers (± SEM) of natural enemies captured in Study 1 in pitfall traps, yellow sticky cards and visually observed in four wheat fields in 2012 and 2013, Michigan State University campus farm East Lansing, Michigan.

Yellow sticky cards captured six families of flying predators. In 2012, in Field 1 the most common family was Dolichopodidae followed by Chrysopidae and Syrphidae while in Field 2 the most common family was Syrphidae followed by Chrysopidae, Dolichopodidae and the coccinellid *Harmonia axyridis*. In 2013, Syrphidae were the most common natural enemies in both fields followed by Nabidae in Field 3, Cantharidae and the coccinellid *Coleomegilla maculata* in Field 4.

During the visual sampling, six natural enemy families were observed. In 2012, *Coccinella septempunctata* and Syrphidae were the most commonly observed in Field 1. In contrast, Araneae were the most commonly observed taxa in Field 2, followed by Coccinellidae larvae and Syrphidae adults. In 2013, the most common taxa were adult *C. maculata* in both fields, following by adult *H. axyridis*, Syrphidae, Carabidae and Anthocoridae in Field 3, and adult *C. septempunctata, H. axyridis* and Syrphidae in Field 4.

### Aphid population growth

**Figure 3:**
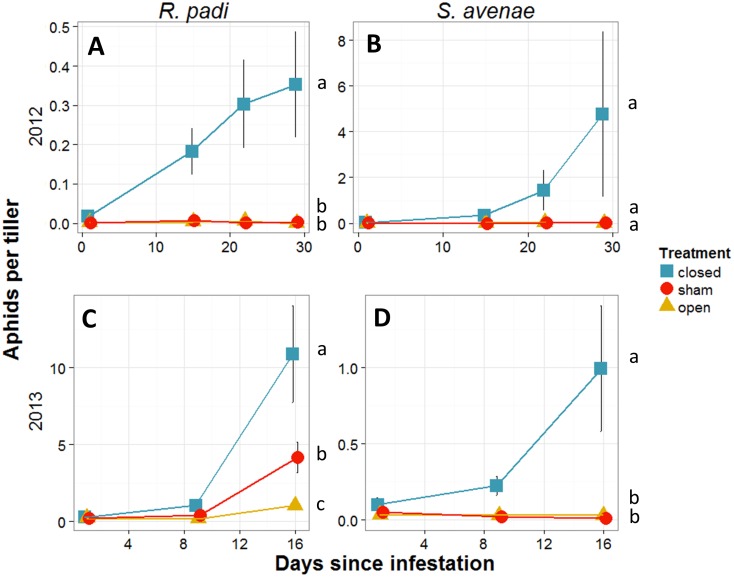
Aphid density, Study 1. Mean aphid numbers (± SEM) per tiller of; **A**) *Rhopalosiphum padi* in 2012, **C**) *R. padi* 2013, **B**) *Sitobion avenae* in 2012, **D**) *S. avenae* 2013, for closed (exclusion of all natural enemies), open (exposed to all natural enemies), and sham (to control for cage effect) plots in Study 1. ANOVA was used to test statistical differences. Different letters next to the treatments indicate statistically significant differences among aphids per tillers at α = 0.05.

Exclusion of natural enemies resulted in increased *R. padi* and *S. avenae* populations in both years (**Figure 3**). In 2012, populations of *R. padi* per tiller were significantly different between treatments (Rao _2, 93_ = 10197, p<0.001) and days (Rao _3, 90_ = 638, p<0.001), and sites were not significantly different from each other (Rao _1, 89_ = 0.8, p = 0.369). In 2013, populations of *R. padi* were significantly different between treatments (Rao _2, 87_ = 4925, p<0.001), days (Rao _2, 85_ = 10493, p<0.001), and sites (Rao _1, 84_ = 2109, p<0.001). In 2012, *R. padi* numbers were lower than in 2013. Despite differences in years and sites, the treatment patterns were the same. Closed plots always had higher *R. padi* per tiller than open and sham. The sham cages were not significantly different from the open cages in 2012, but were significantly different in 2013 due to an intermediate number of *R. padi* in the sham treatment (**Figure 3**).

Populations of *S. avenae* per tiller in 2012 also varied significantly between treatments by ANOVA, (Rao _2, 93_ = 891, p<0.001), days (Rao _3, 90_ = 723, p<0.001), and sites (Rao _1, 89_ = 445, p<0.001) but despite numerical differences between treatments, the Holm-adjusted pairwise t-test did not detect differences between treatment pairs. Populations of *S. avenae* in 2013 were significantly different between treatments (Rao _2, 87_ = 5399, p<0.001), days (Rao _2, 85_ = 204, p<0.001), and sites (Rao _1, 84_ = 91, p<0.001). The closed plots for *S. avenae* contained significantly higher aphid numbers per tiller than sham and open plots, with sham cages not significantly different from open plots (**Figure 3**).

### Study 2: Guild-specific Natural Enemy Impacts

#### Predator sampling

Using all three methods of sampling we captured a total of 4,567 individual natural enemies representing 11 taxa (**Table 3**). No exclusion method completely prevented the occurrence of natural enemies. Pitfall traps captured seven taxa of natural enemies. In the -F-G plots, the most frequently captured taxa were Staphylinidae followed by Carabidae, Araneae and Formicidae. In contrast, in -G, -F, and O plots, the Carabidae were the most common taxon, followed by Araneae, and Staphylinidae. Overall, the numbers of ground-dwelling predators captured in the -F-G and -G plots were less than they were in the -F and O plots.

**Table 3:** Natural enemy captures Study 2.

Collection methods	Plots											
Pitfall traps	-F-G			-G			-F			O		
Coccinellidae	0.1	±	0.0	0.5	±	0.2	0.5	±	0.3	0.9	±	0.3
Carabidae	3.5	±	0.6	4.2	±	0.9	15.4	±	2.0	12.9	±	1.1
Formicidae	1.3	±	0.4	1.9	±	0.5	3.0	±	0.7	1.2	±	0.3
Araneae	2.0	±	0.5	3.8	±	0.5	10.4	±	1.0	11.1	±	1.4
Opiliones	0.1	±	0.0	0.1	±	0.1	0.4	±	0.2	0.1	±	0.1
Elateridae	0.2	±	0.1	0.2	±	0.1	0.1	±	0.1		0	
Staphylinidae	3.7	±	0.9	3.8	±	0.7	8.0	±	1.8	6.5	±	1.2
**Yellow sticky card**												
*C. maculata*		0		0.5	±	0.1	0.4	±	0.1	0.7	±	0.2
*H. convergens*		0		0.03	±	0.0		0			0	
*C. septempunctata*		0		0.1	±	0.1		0		0.03	±	0.03
Other Coccinellidae	0.03	±	0.03	0.2	±	0.1	0.03	±	0.03	0.4	±	0.1
Chrysopidae		0		0.6	±	0.3	0.03	±	0.03	0.6	±	0.2
Syrphidae		0		2.5	±	0.5	0.2	±	0.1	2.6	±	0.5
Nabidae		0		0.1	±	0.1		0		0.3	±	0.2
Cantharidae		0		1.0	±	0.2	0.03	±	0.03	0.7	±	0.3
**Visual observations**												
*C. maculata*	0.7	±	0.2	1.8	±	0.4	0.8	±	0.3	1.6	±	0.4
*C. septempunctata*	0.1	±	0.1	0.1	±	0.1	0.03	±	0.03	0.2	±	0.1
*C. septempunctata* larvae		0		0.2	±	0.1		0		0.3	±	0.1
*H. axyridis*		0		0.1	±	0.0		0		0.1	±	0.0
Other Coccinellidae larvae		0		0.1	±	0.1		0		0.2	±	0.1
Carabidae		0		0.1	±	0.0	0.5	±	0.2	0.4	±	0.2
Syrphidae	0.1	±	0.0	1.9	±	0.4	0.2	±	0.1	0.8	±	0.2
Formicidae	0.2	±	0.1	0.2	±	0.1	0.2	±	0.1	0.2	±	0.1
Araneae	0.2	±	0.1	0.4	±	0.2	0.6	±	0.3	0.2	±	0.1
Opiliones	0.1	±	0.1	0.03	±	0.0	0.1	±	0.0	0.03	±	0.03
Cantharidae		0		0.03	±	0.0		0		0.03	±	0.03
Chrysopidae		0		0.03	±	0.0		0		0.03	±	0.03
Nabidae		0					0.03	±	0.03		0	

Mean number (± SEM) of most abundant natural enemies captured in Study 2 in pitfall traps, yellow sticky cards and by visual observation in wheat study sites in Michigan State University campus, East Lansing, Michigan, 2013. Treatment plots included foliar-predator exclusion (-F), ground-dwelling predator exclusion (-G), all predator exclusion (-F-G) and plots that were completely open to predation (O).

On yellow sticky cards, we captured five families of flying predators. In -F-G plots, a single Coccinellidae was captured. In the -G plots Syrphidae were the most common family captured, followed by Cantharidae. In -F plots, the Coccinellid *C. maculata,* followed by Syrphidae were the most common taxa captured, and in O plots, Syrphidae followed by *C. maculata* and Cantharidae were most frequently captured, together representing more than half of insects captured.

In visual observations, we occasionally observed adult *C. maculata*, *C. septempunctata*, Syrphidae, Formicidae, Araneae and Opiliones in the -F -G plots (i.e. 1–5 individuals of each taxa over the course of the study). In -G plots, *C. maculata* followed by Syrphidae, were most commonly observed, representing almost 75% of observed insects. In the -F plots, *C. maculata* followed by Araneae and Carabidae were most commonly observed, although fewer insects were observed, in general, in these plots. In the O plots, the most common natural enemy was *C.* maculata, at just under 40% of observations, followed by Syrphidae and Carabidae, representing 20 and 10% of observed insects respectively.

**Figure 4:**
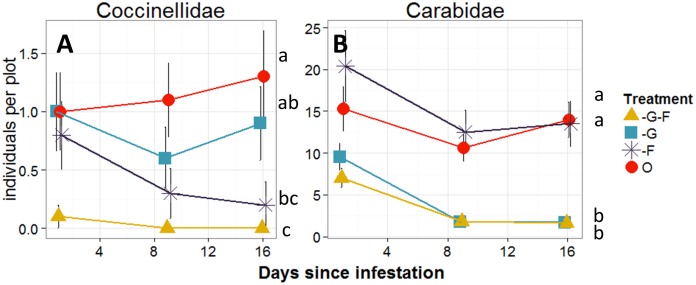
Natural enemy counts. Abundance of **A**) Coccinellidae and **B**) Carabidae captured in sticky cards and pitfall traps, respectively, in -G (excluding ground-dwelling predators), -F-G (excluding all natural enemies), -F (excluding foliar-foraging predators) and O plots (exposed to all natural enemies) in Study 2 (2013). ANOVA was used to test statistical differences. Different letters above the treatments indicate statistically significant differences among aphids per tillers at α = 0.05.

For Carabidae and Coccinellidae, often the most important predators of cereal aphids, the exclusion techniques sufficiently reduced their numbers to allow us to examine their effect on aphid population growth: Coccinellids were significantly reduced in -F-G and -F plots compared to the open plots, and Carabids were significantly reduced in -F-G and -G plots (**Figure 4**).

### Aphid population growth

**Figure 5:**
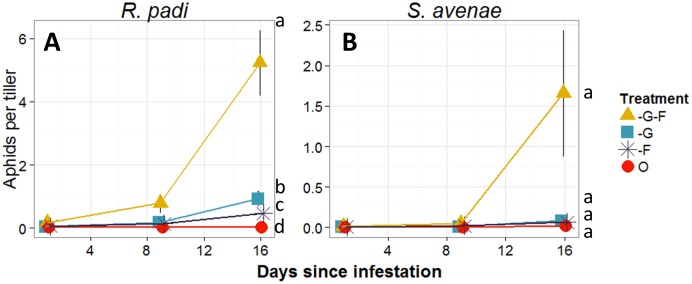
Aphid density, Study 2. Mean number (± SEM) of **A**) *Rhopalosiphum padi*, and **B**) *Sitobion avenae* in 2013 among treatments in Study 2; -F-G (excluding all natural enemies), -G (excluding ground-dwelling predators), -F (excluding foliar-foraging predators) and O (open plot, exposed to all natural enemies). ANOVA with repeated measures was used. For treatment comparisons pairwise t-tests that were Holm-adjusted were used. Different letters indicate statistically significant differences within treatments at α = 0.05.

Treatments manipulating natural enemies had different effects on population growth of *R. padi* and *S. avenae*. *R. padi* populations varied significantly between treatments (Rao _3, 116_ = 4×10^7^, p<0.001), and days (Rao _2, 113_ = 3×10^7^, p<0.001), but not sites (Rao _1, 84_ = 3×10^6^, p = 0.066). In -F-G plots, *R. padi* increased from 0.1 aphids per tiller to a mean of just over 5 aphids per tiller over the three week interval (**Figure 5**). Aphid increase was significantly lower when foliar-foraging predators had access to plots (-G plots). Aphid population increases were even lower when ground-dwelling predators alone (-F plots) had access to plots. Finally, in plots where all natural enemies had access (O plots), aphid populations remained steady at approximately 0.03 aphids per tiller during the 3 week period indicating that the natural enemy community as a whole was very effective in preventing *R. padi* increases.

*S. avenae* populations were lower than *R. padi.* Although ANOVA suggested that populations varied significantly between treatments (Rao _3, 116_ = 159250, p<0.001), days (Rao _2, 113_ = 68233, p<0.001), and sites (Rao _1, 84_ = 233, p<0.001), however, post hoc tests did not find any statistically significant differences, although the general pattern of population growth by treatments was largely similar to that of *R. padi* (**Figure 5**).

## Discussion

From both studies we observed that the intact natural enemy community effectively controlled aphid population densities in wheat in all sites in both years. Similar to other studies in North America [2], [33], [42], [43] the most common taxa of foliar-foraging predators that we found included adult Syrphidae, adult and larval Coccinellidae, adult Chrysopidae, Cantharidae and Nabidae, and the most common ground-dwelling predators were Carabidae, Staphylinidae, Araneae and Opiliones. Unlike other North American studies [44], we observed very low numbers of parasitoid wasps. In addition, the overall abundance of ground-dwelling predators in our system was higher than that of the foliar-foraging predators.

Communities of ground-dwelling and foliar-foraging natural enemies, acting either independently or together, can reduce aphid population densities [25], [26], [30], [45], [46]. In fact, in both of our studies, when both predator groups were present (in the open plots), aphid population growth was very low. This significantly lower aphid population growth provided by the combination of foliar-foraging and ground-dwelling predators suggests a synergy between the different natural enemy foraging groups. Losey and Denno [32] showed the synergistic interaction of ground-dwelling and foliar-foraging predators on pea aphid in alfalfa. The aphids, in response to foraging Coccinelidae, dropped from the alfalfa canopy to the ground where they were consumed by ground-dwelling predators. Our results confirm that in combination these two foraging groups better suppress aphid populations than when acting alone.

Numerous studies show that adult Coccinellidae can suppress cereal aphid populations [42], [47]. It has also been shown that certain Carabidae consume *R. padi* and *S. avenae* [24], [48], [49] and can reduce aphid density [30] by climbing the plant [4] or by preying on aphids when they drop from plants. In our study, ground-dwelling predators alone were more effective than foliar-foraging predators. These results are in contrast to previous European studies [25], [46] where foliar-foraging predators and parasitoids were shown to contribute more to biological control of cereal aphids. Schmidt et al. [25] conducted an experiment in the early-season (May) in Germany and found that parasitoid wasps provided more effective aphid control than ground-dwelling predators. Thies et al. [46] conducted a similar experiment in five European regions and their results suggested that parasitoids and foliar-foraging predators were more important in controlling cereal aphids than ground-dwelling predators, but the relative importance of parasitoids and foliar-foraging predators greatly differed among European regions.

The increased efficacy of ground-dwelling predators that we observed in the present study might be due to the early-season importance of ground-dwelling predators. Previous work has suggested that the effects of ground-dwelling predators on suppressing aphids are strongest in early May [24], [50], [51], when aphid densities in cereals are low and reproduction is slower than in summer. In contrast, flying predators like Coccinellidae which primarily feed on aphids [7] usually become more important once aphid population densities increase [52].

Natural enemies are important for Michigan wheat production because they can keep aphid populations below damaging levels. The average numbers of *S. avenae* we found in the closed plots reached an average of 1–5 aphids per tiller, while in the open plots numbers of *S. avenae* never exceeded 0.03 aphids per tiller on average in either year. The average numbers of *R. padi* in our study reached nearly 11 aphids per tiller in the closed plots in 2013, although it did not exceed 0.3 aphids per tiller in 2012. The open plot reached a maximum average of just over 1 aphid of *R. padi* per tiller in 2013, which is much less than the economic thresholds. The economic threshold in Michigan for both species is 12–15 aphids per tiller [53], although in adjacent regions, economic damage has been observed with aphid numbers as low as 5 per tiller [54].

Under the conditions we studied, natural enemies provided sufficient and reliable aphid suppression, keeping cereal aphid populations below economically damaging levels. It is well established that the use of preventive insecticides may be harmful to existing and effective natural enemy communities [17], [55], and result in pest resurgence [56]. If prophylactic or unwarranted insecticide sprays are used in Michigan wheat, they have the potential to disrupt the ecosystem services provided by the existing natural enemy community. Relying on natural biological control provided by aphid predators and only using chemical control when necessary will help insure more economical and sustainable insect pest management in Michigan wheat.
